# Association of stroke lesion shape with newly detected atrial fibrillation – Results from the MonDAFIS study

**DOI:** 10.1177/23969873221100895

**Published:** 2022-05-25

**Authors:** Bernardo Crespo Pimentel, Thies Ingwersen, Karl Georg Haeusler, Eckhard Schlemm, Nils D Forkert, Deepthi Rajashekar, Pauline Mouches, Alina Königsberg, Paulus Kirchhof, Claudia Kunze, Serdar Tütüncü, Manuel C Olma, Michael Krämer, Dominik Michalski, Andrea Kraft, Timolaos Rizos, Torsten Helberg, Sven Ehrlich, Darius G Nabavi, Joachim Röther, Ulrich Laufs, Roland Veltkamp, Peter U Heuschmann, Bastian Cheng, Matthias Endres, Götz Thomalla

**Affiliations:** 1 Department of Neurology, Medical Center Hamburg-Eppendorf, Hamburg, Germany; 2 Department of Neurology, Christian Doppler Medical Center, Paracelsus Medical University, Salzburg, Austria; 3 Department of Neurology, Universitätsklinikum Würzburg, Wurzburg, Germany; 4 German Atrial Fibrillation Network (AFNET), Münster, Germany; 5 Department of Radiology, University of Calgary, Calgary, AB, Canada; 6 Institute of Cardiovascular Sciences, College of Medical and Dental Sciences, Medical School, University of Birmingham, UK; 7 Departments of Cardiology, UHB and SWBH NHS Trusts, Birmingham, UK; 8 University Heart and Vascular Center Hamburg, Hamburg, Germany; 9 Center for Stroke Research Berlin, Charité-Universitätsmedizin Berlin, Berlin, Germany; 10 Department of Neurology, Universitätsklinikum Leipzig, Leipzig, Germany; 11 Department of Neurology, Martha Maria Hospital, Halle Dölau, Germany; 12 Department of Neurology, University of Heidelberg, Heidelberg, Germany; 13 Department of Neurology, Clinical Center of Hubertusburg, Wermsdorf, Germany; 14 Clinical Center of Hubertusburg, Wermsdorf, Germany; 15 Department of Neurology, Vivantes Klinikum Neukölln, Berlin, Germany; 16 Department of Neurology, Asklepios Klinik Altona, Hamburg, Germany; 17 Department of Cardiology, Universitätsklinikum Leipzig, Leipzig, Germany; 18 Department of Neurology, Alfried Krupp Krankenhaus, Essen, Germany; 19 Department of Brain Sciences, Imperial College London, UK; 20 Comprehensive Heart Failure Center & Clinical Trial Centre Würzburg, University Hospital Würzburg, Germany; 21 Institute of Clinical Epidemiology and Biometry, University Würzburg, Wurzburg, Germany; 22 Klinik und Hochschulambulanz für Neurologie mit Abteilung für Experimentelle Neurologie, Charité-Universitätsmedizin Berlin, Berlin, Germany; 23 German Center for Neurodegenerative Diseases, Partner Site Berlin, Germany; 24 German Center for Cardiovascular Diseases, Partner Site Berlin, Germany; 25 ExcellenceCluster NeuroCure, Berlin, Germany; 26 Berlin Institute of Health (BIH), Berlin, Germany

**Keywords:** Ischemic stroke, cardioembolism, atrial fibrillation, lesion shape

## Abstract

Paroxysmal Atrial fibrillation (AF) is often clinically silent and may be missed by the usual diagnostic workup after ischemic stroke. We aimed to determine whether shape characteristics of ischemic stroke lesions can be used to predict AF in stroke patients without known AF at baseline. Lesion shape quantification on brain MRI was performed in selected patients from the intervention arm of the *Impact of standardized MONitoring for Detection of Atrial Fibrillation in Ischemic Stroke* (MonDAFIS) study, which included patients with ischemic stroke or TIA without prior AF. Multiple morphologic parameters were calculated based on lesion segmentation in acute brain MRI data. Multivariate logistic models were used to test the association of lesion morphology, clinical parameters, and AF. A stepwise elimination regression was conducted to identify the most important variables. A total of 755 patients were included. Patients with AF detected within 2 years after stroke (*n* = 86) had a larger overall oriented bounding box (OBB) volume (*p* = 0.003) and a higher number of brain lesion components (*p* = 0.008) than patients without AF. In the multivariate model, OBB volume (OR 1.72, 95%CI 1.29–2.35, *p* < 0.001), age (OR 2.13, 95%CI 1.52–3.06, *p* < 0.001), and female sex (OR 2.45, 95%CI 1.41–4.31, *p* = 0.002) were independently associated with detected AF. Ischemic lesions in patients with detected AF after stroke presented with a more dispersed infarct pattern and a higher number of lesion components. Together with clinical characteristics, these lesion shape characteristics may help in guiding prolonged cardiac monitoring after stroke.

## Introduction

Cardioembolism accounts for approximately 20% of all ischemic strokes^1,2^ and is mostly caused by atrial fibrillation (AF). Identification of AF following acute ischemic stroke (AIS) or transient ischemic attack (TIA) is crucial as oral anticoagulation is more effective for secondary prevention in this subgroup of patients than antiplatelet agents.^3^ As AF is often clinically silent, it may go undetected during the in-hospital diagnostic work-up, thus depriving a significant proportion of patients of the most efficient preventive treatment.^4^

Different approaches can be used to detect non-permanent AF after stroke. Repeated Holter-ECG is frequently suggested but is not universally available. Invasive cardiac monitoring (ICM) is an ancillary procedure that increases the detection rate of AF^5^ and is suggested for patients without detected AF but high suspicion of a cardioembolic source of stroke.^5,6^ However, there are no established criteria to guide ICM implantation in stroke patients, as there is no randomized controlled trial demonstrating a subsequent reduction of stroke recurrence or death.

Diffusion-weighted imaging (DWI) and fluid-attenuated inversion recovery (FLAIR) have shown high sensitivity in detecting even small ischemic lesions and allow the assessment of complex lesion characteristics such as shape and distribution patterns.^7–9^ The analysis of stroke morphologic features, such as size, lesion dispersion, and sphericity, has been explored in several clinical studies.^7,8,10,11^ Limited evidence from MRI-based studies suggests a potential role of location patterns, such as scattered stroke lesions, cortical involvement, or involvement of multiple arterial territories in anticipating a cardio-embolic source of stroke.^12–17^

Thus, it remains unknown if specific morphologic lesion patterns can be used to identify patients with a high risk of AF. The aim of this pre-defined sub-study of the *Impact of standardized MONitoring for Detection of Atrial Fibrillation in Ischemic Stroke* (MonDAFIS) study^18,19^ was to characterize ischemic stroke lesion shape and location patterns and use these markers to predict a first episode of AF.

## Material and methods

### Study design

In this exploratory, predefined analysis, we included data of patients randomized in the intervention arm of the MonDAFIS study who underwent brain MRI during in-hospital diagnostic work-up. MonDAFIS was an investigator-initiated, randomized, multi-center study conducted at 38 certified stroke units in Germany that assessed the effect of prolonged inpatient Holter ECG monitoring on anticoagulation rates 12 months after AIS/TIA.

Patients with AIS or TIA without known AF before hospital admission were included and randomized 1:1 to standard diagnostic procedures or standard diagnostic procedures plus in-hospital Holter ECG monitoring for up to 7 days. Inclusion criteria included age ⩾18 years and stroke unit admission within 72 h after onset of stroke-related symptoms. Patients with pre-stroke life expectancy less than a year and post-stroke life expectancy less than a month, participation in an interventional study, pregnancy or breast-feeding, independent indication for long-term oral anticoagulation as well as patients with implanted devices with the ability to record an ECG were excluded.^18^

For the current analysis, we further excluded subjects without acute ischemic lesions on DWI, as well as patients with MRI brain imaging acquired later than 7 days following the index stroke or insufficient image quality. In the MonDAFIS study, presence of AF in hospital was defined in accordance with the current *European Society of Cardiology* guidelines as an absolute arrhythmia lasting ⩾30 s.^3^ During the follow-up period of 2 years, detection of AF was assessed through a telephone interview conducted by the study team at the Center for Stroke Research Berlin.

For descriptive and analysis purposes, we divided patients into two groups. In the first group, we included patients who had AF detected in-hospital after the index stroke or TIA as well as patients self-reported to have documented AF within 24 months after the index stroke or TIA. All other study patients were assigned to the second group. All study procedures were carried out in accordance with the Declaration of Helsinki. The study was approved by the ethics committees of Charité – Universitätsmedizin Berlin, Germany, and all participating sites. All patients provided written informed consent.

### Lesion segmentation

Subject datasets were analyzed using a segmentation algorithm (SONIA) based on the in-house developed software tool ANTONIA.^20^ SONIA performs DWI image co-registration to FLAIR images, semi-automated stroke lesion segmentation based on an apparent diffusion coefficient (ADC) threshold, and registration to standard MNI (Montreal Institute of Neurology) space. Ischemic stroke lesions were semi-automatically segmented using an ADC maximum threshold of 620 × 10^−6^ mm²/s. Segmented voxels were then manually corrected by two trained neurology residents (BCP, TI). Each individual lesion mask was registered to the corresponding FLAIR image, being consequently transformed to MNI standard space using an affine registration process. Registration results were visually evaluated and compared with the original lesion in native space as a quality assurance step. Native space masks were further post-processed with removal of implausible lesion components smaller than 10 mm³. MRI data was acquired on 1.5 or 3 T scanners in 20 participating centers. Pixel spacing ranged from 0.71 to 1.88 mm with a slice thickness of 3–6 mm.

### Lesion shape parameters

Morphological descriptors were calculated in lesion masks in native space using customized in-house developed tools. The following brain lesion shape parameters were calculated: (1) number of lesion components; (2) volume of the stroke lesion (mL); (3) surface area of the ischemic lesion (mm^2^); (4) the oriented minimum bounding-box of the lesion (mL, OBB), that is, the smallest rectangular volume comprising all lesion components (whereas the orientation is independent of the coordinate axes); (5) the ratio between lesion volume and OBB volume, that is, the proportion of the OBB filled by the actual stroke lesion; and (6) the normalized shape factor S of the ischemic lesion, which describes how closely the lesion resembles the shape of a sphere, defined as:

$$S=\frac{\frac{\surd A}{\sqrt[{3}]{V}}}{2.199085233}$$

where A is the surface area and V the volume of the lesion.

### Lesion location characterization

Inferences about lesion location were determined using the mindboggle-101 brain atlas^21^ and a previously published vascular territory map.^22^ Location parameters included the affected hemisphere (left, right, both), vascular territory (left/right internal carotid artery, basilar artery, multiple affected territories), and any cortical region affected. A minimum of 100 lesion voxels overlapping the respective location was chosen as a threshold.

## Statistical analysis

Descriptive statistics are reported in absolute counts and percentages or medians and interquartile range, as applicable. Group differences in frequencies were analyzed using the chi-squared test and *p*-values were calculated by Monte Carlo simulation. Groups were compared using the Mann–Whitney test. To determine a potential relation between lesion shape parameters and AF, logistic regression models were fitted in the following way: first, logarithmized parameters were analyzed individually in univariate models. In order to compare the individual impact of different variables, all values were scaled to standardized values (*z*-scores) beforehand. Second, a multivariate model was fitted containing the parameters “number of components,” “lesion volume,” “sphericity,” “OBB volume,” and “ratio between lesion and OBB volume.” Due to collinearity with “lesion volume,” “surface area,” was excluded from the model. This regression model was further refined by a simple stepwise elimination algorithm. During every step, variables that did not reach the significance level of 0.05 were eliminated from the model starting with the least significant variable.

In a second analysis, the multivariate models were complemented by clinical variables (age, sex, NIHSS score at admission) and imaging location parameters (multiple vascular territories affected, cortical involvement). All tests were performed two-sided and statistical significance was set to an alpha of 0.05. Statistical analysis was performed using R Studio (R software package, v. 3.6.2; R Foundation for 159 Statistical Computing, Vienna, Austria).

## Results

**Figure 1.**
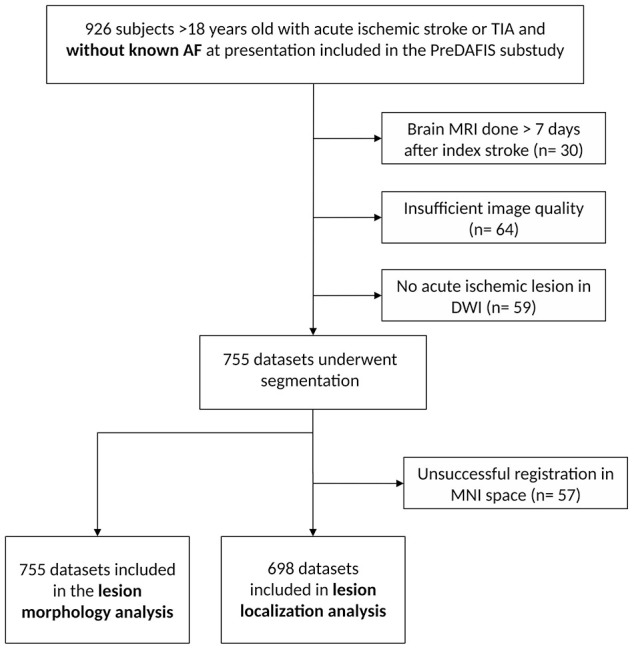
Study flowchart.

We analyzed brain MRI data from 1,054 patients of the MonDAFIS intervention arm (see Figure 1). We excluded patients with missing clinical information (*n* = 146), brain MRI performed >7 days following the index stroke (*n* = 30) and patients without an acute ischemic brain lesion in DWI (*n* = 59). From the remaining subjects, we excluded datasets with insufficient image quality or missing MRI sequences (*n* = 64). Thus, we performed semi-automated segmentation of acute ischemic brain lesions in 755 patients. Information on AF at 24 months after index stroke was missing in 54 patients, which were also excluded. Individual space lesion masks were used for the lesion shape analysis (*n* = 701) and lesion location analysis was carried out in standard MNI space. Due to unsuccessful image normalization to MNI space, 57 datasets were excluded, resulting in 644 subjects available for lesion location analysis.

**Figure 2.**
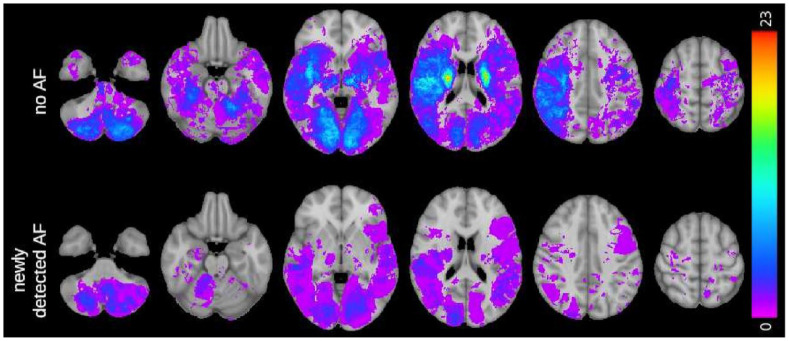
Overlay map of lesion density in patients without detected atrial fibrillation (AF) and with newly detected AF. Lesion density is displayed in the color bar on the right side.

Summary descriptive statistics are displayed in Table 1. Median age was 66 years (IQR 57–76) and 261 patients (37.5%) were female in the shape analysis group (*n* = 701). MRI was performed in a median of 1 day (IQR 1–3 days) after hospital admission. AF was detected in a total of 86 patients (12.3%) within the 24 months follow-up period. Compared to patients without AF, female sex (52% vs 35%, *p* = 0.005) and advanced age (74 years (IQR 67–78) vs 65 years (IQR 56–74), *p* < 0.001) was more often observed in patients with detected AF. No difference regarding the NIHSS score at hospital admission was observed. In the AF group, lesions were more often located in cortical regions (44% vs 28%, *p* = 0.008) (Table 2). Figure 2 shows overlap lesion maps for patients with and without detected AF. The same analysis was done for the cases of in-hospital diagnosed AF (comprising cases detected through monitoring on the stroke unit and normal neurological ward). Due to the low case number of subjects (*n* = 25), these results are displayed as supplemental material (see Supplemental Table 1).

**Table 1.** Group difference in clinical and shape parameters between patients with and without detected atrial fibrillation (AF) within 24 months after ischemic stroke or TIA.

	Total (*n* = 701)	*No AF* (*n* = 615)	*Detected AF* (*n* = 86)	*p*
Clinical variables				
Female sex, *n* (%)	261 (37)	216 (35)	45 (52)	**0.003**
Age, *years* (IQR)	66 (57–76)	65 (56–74)	74 (67–78)	**<0.001**
Admission NIHSS score, *points* (IQR)	2 (1–4)	2 (1–4)	2.5 (1–4)	0.532
Shape parameters				
Lesion components, *n* (IQR)	2 (1–4)	2 (1–4)	3 (1–7)	**0.008**
Lesion volume, *mL* (IQR)	0.39 (0.13–1.13)	0.39 (0.14–1.00)	0.45 (0.11–3.03)	0.336
Surface area, *mm*^2^ (IQR)	39.35 (17.77–79.49)	38.91 (17.86–74.15)	40.34 (16.99–141.5)	0.336
Sphericity, *value* (IQR)	1.26 (1.21–1.32)	1.26 (1.21–1.318)	1.28 (1.22–1.33)	0.319
OBB Volume, *mL* (IQR)	13 (3.37–95.8)	9.87 (3.15–78.3)	58.14 (6.10–180.4)	**0.003**
Lesion/OBB Volume, *ratio* (IQR)	0.05 (0.01–0.13)	0.05 (0.01–0.14)	0.02 (0.00–0.13)	0.108

**Table 2.** Group difference in location parameters between patients with and without detected atrial fibrillation (AF) within 24 months after ischemic stroke or TIA.

	Total (*n* = 644)	No AF (*n* = 565)	Detected AF (*n* = 79)	*p*
Location parameters				
Cortical involvement, *n* (%)	192 (30)	158 (28)	34 (44)	**0.008**
Multiple territories, *n* (%)	42 (6)	35 (6)	7 (9)	0.491
Bilateral involvement, *n* (%)	50 (8)	41 (7)	9 (12)	0.249

Comprehensive lesion shape analysis was carried out in native lesion space. Infarcts in patients with AF had a significantly higher number of separate lesion components (3.0 (IQR 1.0–7.0) vs 2.0 (IQR 1.0–4.0), *p* = 0.008). Additionally, the OBB volume was significantly larger (58 mL (IQR 6.1–180.4) vs 10 mL (IQR 3.2–78.2), *p* = 0.003) in the AF group (see Figure 4).

**Figure 3.**
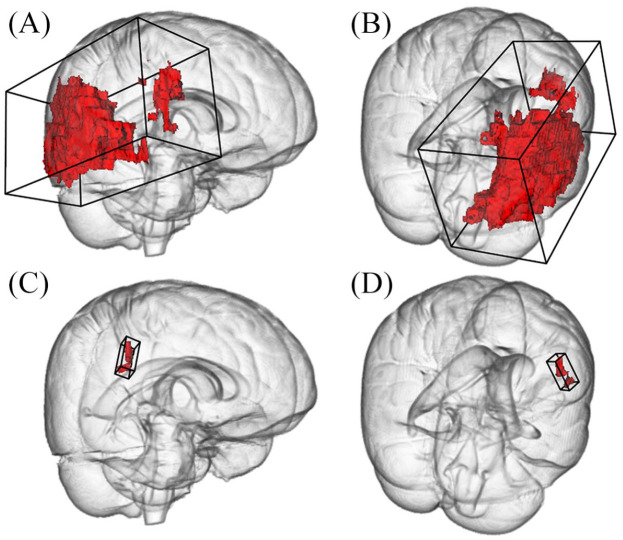
Representative example of two differing right-sided stroke lesions. The large lesion consists of eight components and measures 9.8 mL in total. The resulting overall bounding box (OBB) is 1204 mL in volume (A and B). In contrast, the OBB of a smaller lesion of 0.36 mL consisting of one component is only 7.2 mL in volume (C and D). Three-dimensional reconstructions are depicted from a fronto-lateral (left column) and dorsolateral (right column) view.

In univariate generalized logistic regression models, AF detection within 24 months after the index stroke/TIA was associated with a larger OBB volume (OR 1.42, 95%CI 1.09–1.87, *p* = 0.010) and higher number of lesion components (OR 1.33, 95%CI 1.06–1.70, *p* = 0.013), while no association with lesion volume, surface area, and sphericity was found. In the multivariate analysis including lesion shape characteristics, clinical variables (age, sex, NIHSS at admission), as well as cortical involvement and involvement of multiple vascular territories, only female sex and old age were significantly associated with AF detection (see Table 3). The stepwise elimination algorithm identified age (OR 2.13, 95%CI 1.52–3.06, *p* < 0.001), female sex (OR 2.45, 95%CI 1.41–4.31, *p* = 0.002), and OBB volume (OR 1.72, 95%CI 1.29–2.35, *p* < 0.001) as statistically associated with AF (see Table 3). Figure 3 displays an example of two acute stroke lesions with different OBB volumes.

**Table 3.** Multivariate logistic regression model containing lesion shape parameters and clinical variables and final stepwise elimination model.

	OR (95% CI)	*p*
Multivariate logistic regression		
Age	2.15 (1.53–3.12)	**<0.001**
Female Sex	2.42 (1.38–4.28)	**0.002**
NIHSS score on admission	1.15 (0.88–1.49)	0.297
Cortical involvement	1.41 (0.65–3.05)	0.383
Number of components	1.19 (0.66–2.16)	0.554
Multiple territories	0.71 (0.21–2.08)	0.556
Lesion volume	1.16 (0.71–1.90)	0.562
OBB volume	1.24 (0.58–2.60)	0.579
Sphericity	1.03 (0.73–1.44)	0.855
Stepwise elimination model		
Age	2.13 (1.53–3.06)	**<0.001**
Female sex	2.45 (1.41–4.31)	**0.002**
OBB volume	1.72 (1.29–2.35)	**<0.001**

**Figure 4.**
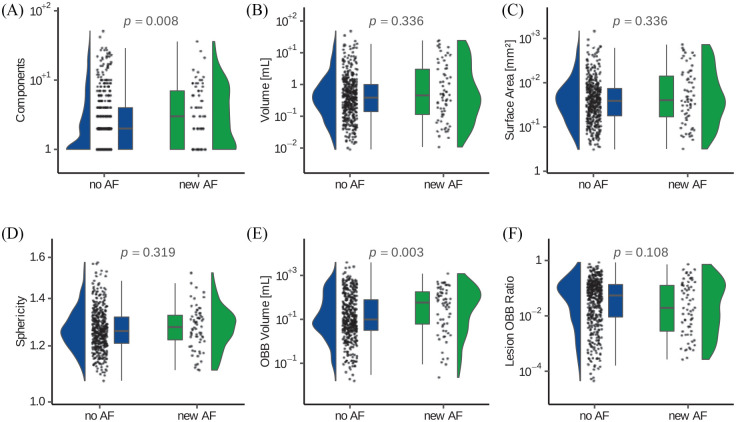
Raincloud and box density plots of the analyzed shape parameters between patients with and without newly detected atrial fibrillation (AF). Lesion volume (B) as well as oriented minimum bounding box (OBB) volume (E) is given in milliliters, while the surface area (C) in mm2.

## Discussion

In this pre-defined analysis of the prospective MonDAFIS study, patients with AF detected within 24 months after AIS showed a higher number of ischemic lesions and a wider spatial lesion distribution in the acute imaging work-up. Older age, female sex, and spatial lesion distribution were independently associated with AF in the analyzed sub-cohort of study patients. The clinical benefit of imaging paramaters should be evaluated in further studies.

In our cohort, stroke patients with detected AF displayed a higher number of lesion components as compared to patients without AF during follow-up. This corroborates results from previous observational studies using MRI-DWI or CT imaging.^11,14,15,23^ In the context of AF, repetitive embolism or large emboli generated in the left atrium that fragment on their way and end up at multiple distinct sites within the cerebral vascular bed likely leads to a multi-site infarct pattern.

Additionally, detection of AF was associated with a significantly larger minimum OBB volume, consistent with a manifold infarct pattern with extensive spatial distribution. In contrast to distal arterial sources of embolic material, cardiac emboli can simultaneously travel through any of the major arteries supplying the brain and may induce a multiple vascular pattern. Indeed, multiple lesion patterns affecting various cerebral vascular territories are associated with a cardio-embolic stroke, a proximal embolic source or the presence of tumors (e.g. in the thoracic aorta).^9,11,24,25^

Moreover, lesions in patients with AF detected during follow-up were of similar size as in patients in whom no AF was detected. This finding contradicts some previous studies demonstrating that ischemic lesions related to AF tend to be larger compared to other etiologies,^24,26^ probably owing to the larger size of cardiac emboli as compared to other sources. The possibility that AF was detected as an incidental finding not etiologically related to the index stroke could have also contributed to the volume similarities.

Cortical involvement in strokes related to AF and other cardiac sources is a matter of conflicting evidence. Supporting the findings of previous studies,^27–29^ cortical involvement was more frequent in patients with detected AF in our cohort. Nevertheless, it was not independently associated with AF in the multivariate regression analysis.

Apart from lesion OBB volume, old age and female sex were identified as significant factors associated detection of AF in the step-wise top-down elimination algorithm applied to both multivariate models. This high predictive value in detection of AF is in line with previous findings.^30,31^

To our knowledge, the HAVOC and AS5F scores^32,33^ are the only clinical scores developed to stratify patients with cryptogenic stroke/TIA into risk categories of AF detection. Both scores are however solely based on clinical and/or ECG data. With this study, we contribute to a growing body of evidence supporting imaging-based scores to identify stroke patients at high-risk of a first episode of AF.

Our study has several limitations. First, the MonDAFIS study was not powered to identify significant predictors for AF in subgroups of patients and a relevant number of designated patients for this subgroup analysis had to be excluded. Despite the relatively large patient cohort, the pragmatic inclusion criteria of the MonDAFIS study constitute a limiting factor, as a substantial number of patients was aged <65 years, and thus, at comparably low risk of AF. Of note, there was no systematic ECG monitoring after hospital discharge. The use of a stepwise regression model may also, despite the small number of variables, favor nuisance variables and therefore affect the robustness of our data.

## Conclusion

Ischemic stroke in patients with detected AF during a 24 months follow-up tend to present with a widespread multiple infarct pattern and a higher number of brain lesions. In addition to old age and female sex, spatial lesion dispersion was independently associated with AF detection. Our results therefore support visual rating of ischemic lesion patterns for AF risk stratification. Whether prolonged ECG monitoring in these patients improves stroke prevention has to further investigated.

## Supplemental Material

sj-docx-1-eso-10.1177_23969873221100895 – Supplemental material for Association of stroke lesion shape with newly detected atrial fibrillation – Results from the MonDAFIS study Click here for additional data file. Supplemental material, sj-docx-1-eso-10.1177_23969873221100895 for Association of stroke lesion shape with newly detected atrial fibrillation – Results from the MonDAFIS study by Bernardo Crespo Pimentel, Thies Ingwersen, Karl Georg Haeusler, Eckhard Schlemm, Nils D Forkert, Deepthi Rajashekar, Pauline Mouches, Alina Königsberg, Paulus Kirchhof, Claudia Kunze, Serdar Tütüncü, Manuel C Olma, Michael Krämer, Dominik Michalski, Andrea Kraft, Timolaos Rizos, Torsten Helberg, Sven Ehrlich, Darius G Nabavi, Joachim Röther, Ulrich Laufs, Roland Veltkamp, Peter U Heuschmann, Bastian Cheng, Matthias Endres and Götz Thomalla in European Stroke Journal
