# Supplementary material for: Association of stroke lesion shape with newly detected atrial fibrillation – Results from the MonDAFIS study
Source: Eur Stroke J. 2022 May 25;7(3):230–7. doi: 10.1177/23969873221100895 (PMC9446317; doi:10.1177/23969873221100895)
Supplement: sj-docx-1-eso-10.1177_23969873221100895 – Supplemental material for Association of stroke lesion shape with newly detected atrial fibrillation – Results from the MonDAFIS study [file sj-docx-1-eso-10.1177_23969873221100895.docx]

**Supplemental List 1.** Participating centers

| Charité Universiätsmedizin Berlin, Berlin Germany |
| --- |
| Klinikum Emden, Emden, Germany |
| Vivantes Klinikum Neukölln, Berlin, Germany |
| Fachkrankenhaus Hubertusburg gGmbH, Wermsdorf, Germany |
| DRK Kliniken Berlin – Köpenick, Berlin, Germany |
| Krankenhaus Martha-Maria Halle-Dölau, Halle, Germany |
| Universitätsklinikum Leipzig AöR, Leipzig, Germany |
| Asklepios Klinik Altona, Hamburg, Germany |
| Universitätsklinikum Heidelberg, Heidelberg, Germany |
| Universitätsklinikum Bonn, Bonn, Germany |
| Universitätsklinikum Hamburg-Eppendorf, Hamburg, Germany |
| Universitätsklinikum Würzburg, Würzburg, Germany |
| Bezirkskrankenhaus Günzburg, Günzburg, Germany |
| MediClin Krankenhaus Plau am See, Plau am See, Germany |
| Universitätsklinikum Schleswig-Holstein - Campus Lübeck, Lübeck, Germany |
| Universitätsklinikum Frankfurt am Main, Frankfurt, Germany |
| Albertinen Krankenhaus, Hamburg, Germany |
| Klinikum Ludwigshafen gGmbH, Ludwigshafen am Rhein, Germany |
| Leopoldina-Krankenhaus der Stadt Schweinfurt, Schweinfurt, Germany |
| Helios Klinikum Erfurt, Erfurt, Germany |

**Supplemental Table 1**. Multivariate logistic regression model containing lesion shape parameters, clinical variables and final stepwise elimination model with AF detected during hospital stay.

|  | **OR (95% CI)** | ***p*** |
| --- | --- | --- |
| Multivariate logistic regression |  |  |
| Age | 2.40 (1.38-4.62) | **0.004** |
| Female Sex | 4.89 (1.87-14.53) | **0.002** |
| NIHSS score on admission | 0.90 (0.51-1.44) | 0.698 |
| Cortical involvement | 1.07 (0.31-3.65) | 0.911 |
| Number of components | 1.55 (0.61-4.04) | 0.360 |
| Multiple territories | 0.31 (0.02-1.92) | 0.289 |
| Lesion volume | 0.88 (0.41-1.94) | 0.757 |
| OBB volume | 1.31 (0.40-4.14) | 0.651 |
| Sphericity | 1.04 (0.57-1.84) | 0.893 |
| Stepwise elimination model |  |  |
| Age | 2.47 (1.41-4.71) | **0.003** |
